# Application and challenge of pancreatic organoids in therapeutic research

**DOI:** 10.3389/fphar.2024.1366417

**Published:** 2024-05-24

**Authors:** Jin Chen, Jin Lu, Shu-Na Wang, Chao-Yu Miao

**Affiliations:** ^1^ Department of Endocrinology and Metabolism, Changhai Hospital, Second Military University /Naval Medical University, Shanghai, China; ^2^ Department of Pharmacology, Second Military Medical University /Naval Medical University, Shanghai, China

**Keywords:** pancreatic organoids, human organoids, modeling pancreatic diseases, drug screening, regenerative medicine

## Abstract

The *in-vivo* non-human primate animal and *in-vitro* cell disease models play a crucial part in the study of the mechanisms underlying the occurrence and development of pancreatic diseases, but with increasingly prominent limitations with in-depth research. Organoids derived from human pluripotent and adult stem cells resemble human *in-vivo* organs in their cellular composition, spatial tissue structure and physiological function, making them as an advantageous research tool. Up until now, numerous human organoids, including pancreas, have been effectively developed, demonstrating significant potential for research in organ development, disease modeling, drug screening, and regenerative medicine. However, different from intestine, liver and other organs, the pancreas is the only special organ in the human body, consisting of an exocrine gland and an endocrine gland. Thus, the development of pancreatic organoid technology faces greater challenges, and how to construct a composite pancreatic organoid with exocrine and endocrine gland is still difficult in current research. By reviewing the fundamental architecture and physiological role of the human pancreas, along with the swiftly developing domain of pancreatic organoids, we summarize the method and characteristics of human pancreatic organoids, and its application in modeling pancreatic diseases, as a platform for individualized drug screening and in regenerative medicine study. As the first comprehensive review that focus on the pharmacological study of human pancreatic organoid, the review hopes to help scholars to have a deeper understanding in the study of pancreatic organoid.

## Introduction

The *in-vivo* animal and *in-vitro* cell models play a crucial role in studying the occurrence, development, prevention and treatment of pancreatic disease (Casamitjana et al., 2022; Yumi et al., 2023). Traditional animal models such as mouse have significant differences in cellular, genetic, immune, and pharmacokinetic features due to species differences with humans, resulting in poor predictive ability for actual human responses. Non-human primates, on the other hand, are expensive and difficult to perform high-throughput screening, resulting in limited applications. In the past decade, the development of humanized pancreatic organoids has made up for the limitations for traditional research models. Organoids are the *in-vitro* formed three-dimensional tissues derived from adult stem cells (ASCs) or pluripotent stem cells (PSCs), which have highly similar tissue properties and physiological functions to the corresponding *in-vivo* organs (Marsee et al., 2021). Hans Clevers lab., in 2009, reported that *in-vitro* induction of murine LGR5^+^ intestinal stem cells into intestinal organoids featuring intestinal crypt-villus formations is possible (Lancaster and Knoblich, 2014). Intestinal organoids derived from human pluripotent stem cells (hPSCs) and clinical primary ASCs were developed in 2011. In 2013, organoids of liver, kidney and pancreas which developed from hPSCs were successfully cultivated. In 2014, the concept of organoids was first systematically proposed.

There are several advantages of human organoids. On the one hand, human organoids not only could represent *in-vivo* human physiology, but also can be quickly and easily obtained from healthy individuals or patients. Currently, human organoids are mainly produced by hPSCs and clinical primary ASCs. In addition to derived from healthy tissues, it is also possible to extract organoids from patient tissues for disease modeling, such as tumor organoids. And both of them are capable of replicating the physiological and pathological conditions in human organ tissues (Clevers, 2016). On the other hand, human organoids are stable, capable of large-scale genome screening and drug screening on plates, and most emerging genetic engineering tools can be introduced at the level of pluripotent stem cells or straightly into organoid systems. The disease model can be prepared by using gene editing technology to modify normal human stem cells or directly isolating diseased tissue (Xinaris et al., 2015). Nowadays, organoid technology has been relatively mature application in the study of a variety of diseases. Currently, a range of human organoids, including brain (Mansour et al., 2018), liver (Takebe et al., 2017), intestine (Fujii et al., 2018), and kidney (Dilmen et al., 2023) have been successfully demonstrated significant promise in the fields of organ development, drug screening and regenerative medicine study. And, more attention has been paid to develop models *in vitro* for the 3D culture systems of human pancreas.

The pancreas is the special organ in the human anatomy. During the embryonic period, the pancreas sprouts from the primitive foregut endoderm of the embryo, and develops into pancreatic tissue. The pancreas is a very important digestive organ in human body, which is composed of endocrine and exocrine compartments. The exocrine pancreas accounts for over 90% pancreas’s total volume, containing about 5% ductal and 85% acinar epithelia. A range of digestive enzymes secreted by acinar cells enter the duodenum through the pancreatic duct and choledoch. In addition to secreted water and bicarbonate, pancreatic stem cells are found in the ductal epithelial cell region (Lee et al., 2012). Endocrine precursor cells differentiated from ductal epithelial cells migrate to the pancreatic matrix and eventually differentiate into islet. Islet is organized as a core of β cells encircled by a layer of α, δ, and pancreatic polypeptide (PP) cells. The islet is responsible for controlling blood glucose levels, leading to the production of insulin, somatostatin, glucagon, and pancreatic polypeptide. When pancreatic exocrine function is insufficiency, it usually shows digestive-related diseases, such as acute and chronic pancreatitis, and pancreatic cancer. Pancreatitis is a common disease in the pancreatic exocrine disease, which correlates with significant illness, death rates, and economic burden in the world (Peery et al., 2019). Observational studies have shown that individuals suffering from both acute and chronic pancreatitis face a heightened risk of developing pancreatic ductal adenocarcinoma (PDAC) (Gandhi et al., 2022). There has been a steady rise in both the frequency and mortality attributed to pancreatic tumors from 1990 to 2019 (Kan et al., 2023). Even with advancements in identifying and treating pancreatic cancer, only about 11% patients of pancreatic cancer may persist for a duration of 5 years after diagnosis (Siegel et al., 2022). In addition to surgical treatment, there is also chemotherapy for pancreatic cancer patients. But individual pancreatic cancer patients have different reactions to chemotherapy drugs, and the drug resistance phenomenon is common with non-ideal overall therapeutic efficacy (Peery et al., 2019). In order to provide more effective treatment for cancer patients, medical workers have proposed precision oncology which is a new and personalized medical strategy that develops the most appropriate personalized treatment plan based on individual genetic analysis and tumor molecular characteristics (Picozzi, 2024).

Diabetes mellitus, as the main manifestation of pancreatic endocrine insufficiency, is one of the common metabolic diseases posing a risk to human health. Long-term insulin injections as well as diet restrictions greatly reduce the standard of living among patients. As the social and economic levels persistently advance, the prevalence of type 2 diabetes mellitus (T2DM) has increased globally and shows popular trend in younger people (Henning, 2018). The incidence of diabetes is growing every year. The occurrence rate of T2DM in China was at 12.4% in 2018, and its complications have become a significant burden on the health of Chinese people (Xue et al., 2022). Patients diagnosed type 1 diabetes mellitus (T1DM) need to inject insulin daily to control their blood glucose. For patients, regenerating their own β cells with function of insulin secretion may be an effective and safe solution without strict use of immunosuppressive agents.

Although the technology of pancreatic ductal organoids and pancreatic islet organoids has been increasingly optimized (Merz et al., 2023). Currently, there is no comprehensive review on the pharmacological application of pancreatic organoids. How to establish a complete pancreatic organoid containing both exocrine and endocrine glands still needs to be explored in the future. Thus, by reviewing the rapidly evolving field of pancreatic organoids, this review mainly summarizes the therapeutic application of pancreatic organoids in the pharmacological study of modeling human pancreatic diseases, as a platform for personalized drug evaluation, and application in regenerative medicine study.

## The methods for generating pancreatic organoids and their characterization

### Organoids of pancreatic exocrine gland

Organoids have extremely high potential for application in pancreatic development and the establishment of disease models. hPSCs are expected to be sources of producing various cell types *in-vitro* culture (Sharma et al., 2020). Pancreatic progenitors possess the capability to differentiate into acinar cells, endocrine cells and ductal cells (Mamidi et al., 2018). Pancreatic progenitors can be produced by hPSCs by following the sequential induction of endoderm, foregut endoderm, and pancreatic endoderm (Xie et al., 2013; Kao et al., 2015; Teo et al., 2015). Numerous research works have documented the capacity to guide pancreatic progenitors originating from hPSCs towards the exocrine lineage, endorsing the creation of organoid structures resembling pancreatic ducts or acini (Huang et al., 2015; Simsek et al., 2016; Hohwieler et al., 2017). The progenitor cells of all pancreatic epithelia exhibit pancreatic and duodenal homeobox 1 (PDX1) expression. PDX1^+^ pancreatic progenitors cells have been employed in the production of ductal and acinar lineage-dedicated organoids within matrigel-based culture framework (Pagliuca et al., 2014). Breunig and colleagues (Breunig et al., 2021) have developed a method of pancreatic duct-like organoids (PDLOs) derived from hPSCs *in-vitro*. WNT signaling (Pasca di Magliano et al., 2007) plays a crucial role in embryonic development (Murtaugh et al., 2005; Heiser et al., 2006). Huang et. al have also obtained progenitors of human pancreas, originating from human embryonic stem cells (hESCs), underwent systematic examination of various factor and small molecule combinations, refining a procedure to trigger the transformation of pancreatic progenitors into organoids specific to ductal or acinar lineages. The identification of duct-like organoids is facilitated through the stimulation of fibroblast growth factor, epidermal growth factor, unconventional WNT family, retinoic acid routes, and the suppression of histone deacetylase (HDAC), standard WNT family, and ALK5 pathways. Conversely, the differentiation of acinus organoids is enhanced through the stimulation of the standard WNT, fibroblast growth factor, and cortisol routes, along with the suppression of the hedgehog, NOTCH family, bone morphogenetic protein, and ALK5 pathways (Huang et al., 2015). Individualized models of pancreatic dysplasia and carcinogenesis *in-vitro* are limited by inadequate differentiation of hPSC into pancreatic exocrine lineage. Kleger and colleagues developed a differentiation protocol guiding hPSCs into functional PDLOs, and showed that the PDLO system is suitable for *in-vitro* and *in-vivo* disease modeling on early pancreatic tumor formation. Each oncogene causes a specific growth, structure, and molecular phenotype of PDLOs *in-vitro.* While transplanted PDLOs with oncogenic KRAS alone form heterogeneous dysplastic lesions or cancer, the combination of KRAS with CDKN2A loss leads to the development of dedifferentiated pancreatic ductal adenocarcinomas. In contrast, transplanted PDLOs with mutant GNAS result in the formation of intraductal papillary mucinous neoplasia-like structures (Breunig et al., 2021). Several researches suggest the presence of a progenitor pool for the pancreas in the ductal tree of an adult pancreas (Criscimanna et al., 2011; Furuyama et al., 2011; Kopp et al., 2011) and also endocrine lineages (including β cells) (Xu et al., 2008; Criscimanna et al., 2011; Pan et al., 2013; Van de Casteele et al., 2013). Lgr5 marks the ASCs in various adult organs and acts as a receptor for the WNT-agonistic R-spondins to form pancreatic organoids (Huch et al., 2013). Pancreatic ducts containing Lgr5 stem/progenitor cells can be clonically expanded and eventually acquire continuous proliferating typical pancreatic organoids originating from ductal cells. And based on this theory, pancreatic organoids can also originate from both healthy human tissues and tumor tissues (Bhalerao et al., 2023). Human tumor organoids can be cultured from patient tumor tissues by clinical endoscopic biopsies. Moreover, histology and phenotypic heterogeneity were conserved in tumor organoids (Huang et al., 2015).

### Organoids of pancreatic endocrine gland

**FIGURE 1:**
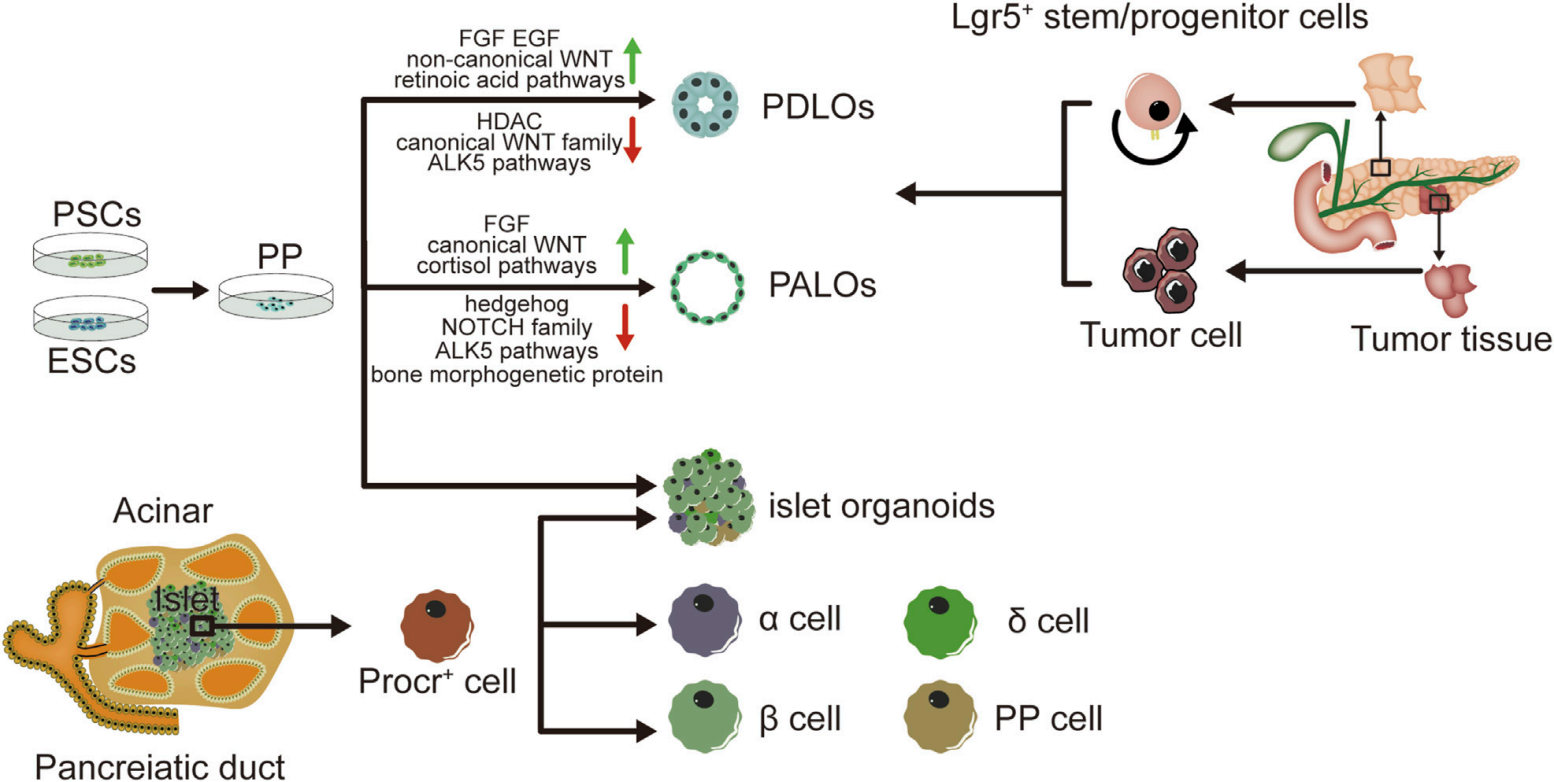
Graphical summary of the method and characteristics in pancreatic organoids. Pancreatic organoids produced by hPSC, human embryonic stem cells hESC, human normal tissues and tumor tissues. Incorporation of human amniotic epithelial cells hAEC into islet organoids markedly enhances engraftment, viability and graft function in a mouse type 1 diabetes model. hPSC: human pluripotent stem cell; hESC: human embryonic stem cell; hAEC: human amniotic epithelial cells; PDLOs: pancreatic duct-like organoids; PALOs: pancreatic acinar like organs; PP: pancreatic progenitors.

With the development of stem cell technology, β cells can be induced from hESCs and hPSCs. Pancreatic endoderm cells (expressing marker genes PDX1, FOXA2, HNF6, NKX6.1) induced from hESCs can be differentiated into endocrine cells after transplantation into mice, and the mature grafts can release insulin and improve blood glucose levels in diabetic model of mice (Kroon et al., 2008; Bruin et al., 2013; Shapiro et al., 2021). However, the cells obtained from hESCs are more like embryonic pancreatic islet cells than adult pancreatic islet cells, and are unable to consistently express mature β cells marker genes (Bruin et al., 2014; Hrvatin et al., 2014). The proteomic analysis of rat pancreatic extracellular matrix hydrogels revealed collagen V (ColV) as a pivotal regulator of islet organogenesis in hPSCs. In the presence of ColV niches, there was a significant upregulation in the expression levels of key pancreatic transcription factors and major hormone genes in iPSC-derived organoids. The presence of ColV in the microenvironment significantly enhanced the glucose-responsive secretion of both insulin and glucagon hormones from organoids (Bi et al., 2020). Transplantation of pancreatic islet cells derived from hPSCs holds great promise as a potential treatment for diabetes. A study has demonstrated the characterization of functional properties in the generation of stem cell derived islets (SC-islets). By employing an optimized the protocol, it is possible to generate human SC-islets that exhibit glucose-sensitive insulin release and endocrine cell composition that is similar to primary islets. Furthermore, through comprehensive functional assays, cellular physiology analyses, metabolic tracing experiments, and single-cell RNA transcriptomic data collected over a period of 6 weeks during *in-vitro* maturation and 6 months following mouse engraftment, this study reveals the temporal acquisition of metabolic programs and gene regulatory changes that contribute to β cell functional maturation (Balboa et al., 2022). Meanwhile, it has been discovered that pancreatic endoderm cells derived from implanted PSCs emit glucose-reactive C-peptide in individuals with T1DM (Ramzy et al., 2021; Shapiro et al., 2021). The achievement of long-term euglycemia following intraportal islet transplantation is hindered by significant peri-transplant islet loss due to inflammation, ischemia, and inadequate angiogenesis. Incorporating human amniotic epithelial cells (hAECs) into islet-cell constructs can enhance resistance against ischemia, expedite revascularization, and promote recovery of cell-to-matrix interactions. Consequently, the inclusion of hAECs in islet organoids significantly improves engraftment as well as the survival and functionality of grafts in a mouse of T1DM (Lebreton et al., 2019). Huang et al. have described a method for transforming human gastric stem cells into pancreatic islet organoids which are similar to β-cells in molecular features and functionality. Stomach-derived human insulin-secreting organoids restore glucose balance in diabetic mice for over 100 days after transplantation(Huang et al., 2023). Research has demonstrated that insulin-reactive cells possessing paneth/goblet cell characteristics can transform into insulin lineages following the ablation of forkhead box O1, either genetically or pharmacologically (Du et al., 2022). It has been documented that the expansion of adult human pancreatic tissue is possible through 3D organoids, and these organoids can be grown over time. Embedded organoids evolved into INS^+^ cells, and the presence of circulating human C-peptide was identified during a glucose test at 1 month of post-transplantation in the beneath the kidney capsule of immunodeficient mice (Loomans et al., 2018). Zeng Yi’s team identified precursor cell lines in adult pancreas that differentiate into four types of endocrine cells, and cultured them to become functional organoids with mature β cells. Single-cell RNA sequencing reveals the presence of Procr^+^ cells in the pancreas of adult mice. Situated in the islets, these cells lack markers of differentiation, and have endothelial to mesenchymal properties. Procr^+^ islet cells have ability to produce cell clones in an adult environment and differentiate into four endocrine cells by genetic lineage tracing. The sorted Procr^+^ cells constitute less than 1% of the total islet cells, but can form islet organoids when the cells form a clonal density. Serial passage of cells can maintain exponential expansion and induce differentiation of cells at any time point of *in-vitro* culture. Applying the culture system to human cells necessitates additional research. Investigating this ancestral group could reveal their presence in humans and their potential in managing diabetes. β cells accounted for the main component, while α, δ and PP cells accounted for a relatively small proportion in islet organoids. Islet organoids can react with glucose and produce insulin (Wang D. et al., 2020). As mentioned before, adult ductal cells are also believed to acquire embryonic characteristics and produce endocrine cells under special conditions. As an indicator for adult stem cells in multiple organs, Lgr5 is expressed in injured and regenerated pancreatic ducts, while Lgr5^+^ duct cells can produce pancreatic exocrine organoids *in vitro* and differentiate into ducts and endocrine cells after transplantation induction (Huch et al., 2013). A peptide originating from the extracellular matrix determines the destiny of induced PSCs, leading to the creation of endocrine progenitors and later islet organoids (Heaton et al., 2023) (see Figure 1).

### Pancreatic organoids containing exocrine and endocrine gland

Although technologies of pancreatic ductal and pancreatic islet organoids have achieved some success, it remains to be explored how to construct a complete pancreatic organoid system with both endocrine and exocrine gland and function. Many pancreatic diseases often involve both the endocrine and exocrine parts simultaneously. Therefore, it is imperative to construct pancreatic organoids with multi-lineage differentiation potential of endocrine and exocrine gland. One research pioneered the method of directing hPSCs towards multipotent pancreatic progenitors through the examination of singular pancreatic progenitor cells enriched with glycoprotein-2 (Merz et al., 2023). This establishes the foundation for future pancreatic organoids. Besides, the concept of assembloid has been proposed in recent years by putting together two or more different organoids (Pasca et al., 2022). It remains to be studied whether pancreatic assembloid (namely, the complete pancreatic organoid) can be obtained by *in-vitro* fusion culture of pancreatic exocrine and endocrine organoids.

### The application of pancreatic organoids in modeling pancreatic disease Pancreatic ductal adenocarcinoma

Expression of the PDAC associated oncogene GNASR201C was found to induce cystic development in ductal organoids is more efficient than in acinar organoids, whereas KRASG12D is found to be expressed in acinar was more effective in mimicking cancer *in vivo* than in ductal organoids. This demonstrates the lineage tropism and plasticity of genetic action in human pancreatic cancer (Huang et al., 2021). Each oncogene can cause specific growth, structural, and molecular phenotypes *in-vitro*. As mentioned above, hPSC expressing carcinogenic GNAS or KRAS can differentiate into PDLO characterized by the morphological, transcriptional, proteomic, and functional traits of human pancreatic ducts. Cultured PDLO with carcinogenic KRAS alone formed heterodysplastic lesions or cancers, but KRAS with CDKN2A deletion developed into dedifferentiated PDAC (Breunig et al., 2021). Orthotopic transplant organoids can review the development of PDAC tumors at all stages. Another research conducted single-cell RNA sequencing on corresponding primary tumors and organoids from one cholangiocarcinoma and two PDAC patients, seeking to reveal the similarities and differences between primary tumors and their respective organoids on a transcriptome scale (Chen et al., 2023). The research discovered that ST6GAL1 levels are elevated in individuals with initial-stage PDAC, with additional increases noted in later stages of the disease. The validity of this finding has been confirmed through experiments on acinar cells and organoids in mice that show transgenic ST6GAL1 expression (Bhalerao et al., 2023).

Through extensive transcriptomic study of human PDAC organoids, the behavior of organisms is linked to gene expression subtypes. By refining organoid culture conditions, Takashi Seino et. al established 39 patient-derived PDACs and conducted an extensive molecular analysis, shedding light on various forms of WNT/R-spondin niche reliance linked to different gene expression variants. Organoids derived from healthy human ducts have been employed as models for pancreatic cancer. Furthermore, The CRISPR-Cas9-driven modification of pancreas organoids revealed a gradual development of PDAC, progressively becoming independent of their niches. The use of overexpression vectors has been instrumental in researching the initiation and development of PDAC (Seino et al., 2018). Cells extracted from surgically removed tumors have undergone organoid culturing, based on established protocols with some alterations. The primary tumors underwent exome sequencing. Studies have identified somatic mutations of PDAC in a variety of genes played roles in signal transduction routes, epigenetic alterations, genome upkeep, and metabolic enzymes. One potential focus was integrin-linked kinase, and it was verified that an integrin-linked kinase inhibitor could inhibit the growth of organoids derived from patients (Shiihara et al., 2021). By co-culturing PDAC cells from patients with mesenchymal and vascular endothelial cells from hiPSCs, a combined pancreatic cancer organoid was developed, beneficial for researching PDAC recurrence (Takeuchi et al., 2023).

### Intraepithelial neoplasia of pancreatic duct and pancreatic of cystic fibrosis

Pancreatic intraepithelial neoplasia (PanIN) is a continuous process that describes the progression of atypical proliferation of epithelial cells in various levels of pancreatic ducts (including the main pancreatic duct) to carcinoma *in situ*. Divided into three levels of PanIN, PanIN-1 belongs to low grade, while PanIN-3 belongs to precancerous lesions. Using organoid culture, Sinha et. al demonstrated that sensory neurons encouraged the widespread growth of PanIN organoids via substance P receptor neurokinin 1-R signaling and STAT3 activation (Sinha et al., 2017). PDLO with mutant GNAS gene results in intraductal papillary mucinous tumor-like structure (Breunig et al., 2021). Compared to standard murine organoids, mouse PanIN organoids showed a recombination of the conditional KRAS-LSL-G12D allele and increased KRAS-GTP levels (Matsuura et al., 2020). The outcomes of RNA sequencing were verified the increased expression of Agr2, Acsm3, Gcnt1, Gcnt3, and Ugdh, along with the decreased expression of the Ptprd gene in mouse PanIN and mouse Tumor organoids. The pancreatic elements of cystic fibrosis, a genetic disorder, result from mutations, either nonsense or missense, in the cystic fibrosis transmembrane conductance regulator (CFTR) gene. A novel organoid system based on PSCs, employed in simulating the pancreatic elements of cystic fibrosis. *In-vitro*, pancreatic organoids from cystic fibrosis patients mimic impaired CFTR activity, enabling further drug testing and mRNA-driven gene enhancement (Hohwieler et al., 2017). At present, there is no research on human PanIN organoids, and future study will further focus on related studies.

### Diabetes mellitus

Lyu et. al analyzed examined the 3D structure of chromatin during the transformation of hESCs into pancreatic islet organoids. CTCF and cohesion play an important role in regulating gene activity throughout cellular differentiation (Lyu et al., 2023). By jointly cultivating islet and mesenchymal stromal cells, Gooch et. al developed islet-sized organoids, known for their potent immune and cyto-protective, anti-inflammatory, proangiogenic. Simultaneously, researchers have found that administering islet-sized organoids in the omentum via allogeneic, intraperitoneal injection successfully restored lasting normal blood glucose levels in NOD mice with autoimmune diabetes, without suppressing the immune system (Gooch et al., 2023). Another research reported an innovative optogenetic control mechanism for insulin release in pancreatic islet organoids derived from hPSCs, employing monster-opto-Stromal interaction molecule 1. After transplantation, diabetes mice produced human C-peptide (Choi J. et al., 2023). The HIF-1 α Inhibitor PX-478 targets human pancreatic islet organoids subjected to prolonged high glucose stimulation, enhancing insulin production (Ilegems et al., 2022). T2DM represents a structured metabolic disorder affecting multiple organs, marked by the active interaction between these organs. A new microfluidic multi-organoid system can co-culture liver and islet organoids derived from hiPSCs for a duration of up to 30 days. The co-culture system facilitates the delicate release of insulin stimulated by glucose from islet organoids and enhances glucose consumption in liver organoids through glucose tolerance assessments. Metformin is capable of mitigating mitochondrial malfunction and reducing the glucose transport ability of liver and islet organoids under high glucose condition (Tao et al., 2022). Juvenile-onset diabetes typifies Wolfram syndrome, a genetic disorder inherited through WFS1 mutations (Rohayem et al., 2011). Pancreatic β cells, derived from iPSCs of Wolfram syndrome sufferers, exhibited reduced insulin levels and heightened molecular activity due to endoplasmic reticulum stress, suggesting WFS1 deficiency as the cause of β cell failure (Shang et al., 2014; Maxwell et al., 2020).

The application of pancreatic organoids in modeling pancreatic disease can be seen in Table 1. And in the future, pancreatic organoids are anticipated to study the pathogenesis in more pancreatic disease models.

**TABLE 1:** The application of pancreatic organoids in modeling pancreatic diseases.

Cell sources	Disease model	Application	References
hPSCs	PDAC	The lineage tropism and plasticity of genetic action in human pancreatic cancer	Huang et al. (2021)
hPSCs	PDAC	KRAS with CDKN2A deletion developed into dedifferentiated PDAC	Breunig et al. (2021)
Human Pancreatic Tumor	PDAC	The CRISPR-Cas9-driven modification of pancreas organoids revealed a gradual development of PDAC	Seino et al. (2018)
Human Pancreatic Tumor	Cholangiocarcinoma and PDAC	Reveal the similarities and differences between primary tumors and their respective organoids on a transcriptome scale	Chen et al. (2023)
Human Pancreatic Tumor	PDAC	ST6GAL1 was upregulated in patients with early-stage PDAC and was further increased in advanced disease	Bhalerao et al. (2023)
Human Pancreatic Tumor	Pancreatobiliary cancer	Integrin-linked kinase was one candidate target	Shiihara et al. (2021)
Human Pancreatic Tumor	PDAC	Create a fused pancreatic cancer organoid which would be useful for studying PDAC recurrence	Takeuchi et al. (2023)
Mouse	PanIN	Sensory neurons encouraged the widespread growth of PanIN organoids	Sinha et al. (2017)
Mouse	PanIN	KRAS play an important role in murine PanIN organoids	Matsuura et al. (2020)
hPSCs	Model pancreatic aspects of cystic fibrosis	Study on the Mechanism of Pancreatic Cystic Fibrosis	Hohwieler et al. (2017)
Human islet	DM	Improve blood glucose	Gooch et al. (2023)
hPSCs	DM	Pancreatic islet organoids produced human c-peptide	Choi et al. (2023b)
Human islet organoids	DM	Antidiabetic therapeutic agent	Ilegems et al. (2022)
iPSCs	Wolfram syndrome	Research on the Mechanism of Wolfram Syndrome	Shang et al. (2014), Maxwell et al. (2020)

hPSCs: human pluripotent stem cells; PDAC: pancreatic ductal adenocarcinoma; iPSCs: induced pluripotent stem cells; DM: diabetes mellitus.

### Pancreatic tumor organoids as a platform for personalized drug screening

Organoids of pancreatic cancer originating from patients display characteristics of the original tumor, potentially serving as an effective preclinical instrument for forecasting drug reactions. For majority of patients, there is no targeted treatment option for PDAC, and available therapies are limited by toxicity. Pancreatic cancer organoid library can be achieved through patients with suspected or confirmed pancreatic cancer. By testing single drugs on pancreatic cancer organoid library, it offers the benefit lies in choosing new mixes of drugs with high potential, steering clear of substances that are ineffective and thus toxic. Evaluation of patient-derived organoids has been explored as a biomarker for treatment response and individualized treatment in patients with pancreatic cancer, which is capable of forecasting how patients with pancreatic cancer react to neoadjuvant. Establishing organoids derived from patients, both in those new to chemotherapy and after neoadjuvant treatment, facilitates the generation of patient-derived organoids over time to evaluate the evolving sensitivity profiles of chemotherapy. Rapid screening of patient-derived organoids may aid in the early categorization of patients into the most effective neoadjuvant treatment (Demyan et al., 2022). The objective of the HOPE trial (Harnessing Organoids for Personalized Therapy) is to proactively create organoids derived from PDAC patients and assess their responsiveness to medication and its correlation with clinical results. This study confirms that classifying patient-derived organoid as sensitive or resistant to chemotherapy regimens can predict clinical outcome in study subjects (Hossan et al., 2023). Alteration in the cyclin dependent kinase inhibitor 2A (CDKN2A) is very common in PDAC. Chemosensitivity analysis of PDAC cell lines and organoids derived from patients revealed a link between the inactivation of CDKN2A and heightened responsiveness to paclitaxel and SN-38 (Lin et al., 2020). Carfilzomib, a proteasome inhibitor, has demonstrated encouraging outcomes in preliminary research., but not well in clinical applications. By testing the proteasome inhibitor carfilzomib sensitive PDAC derived primary cell culture (PDPCC) subpopulations, a transcriptomic signature indicative of carfilzomib chemosensitivity was chosen through independent component analysis of the PDPCC transcriptome. This feature was validated in an independent cohort of pancreatic organoids derived from PDAC biopsy. The findings demonstrate that the presence of carfilzomib sensitive PDACs with specific transcriptome phenotypes can explain the biological reasons for this response (Fraunhoffer et al., 2020). The study identified the spatial arrangement of organoids that monitor resistance to subclonal chemotherapy in cases of pancreatic and ampullary cancer (Hossan et al., 2023). Integrin-linked kinase inhibitors have been shown to inhibit the expansion of organoids originating from patients. By integrating exome sequencing with organoid culture, it is possible to pinpoint specific targets for individualized medications and evaluate the efficacy of these agents in organoids (Shiihara et al., 2021). Beutel et. al discovered that the immediate isolation, spread, and pharmaco-typing of organoids from pancreatic cancer patients could facilitate the prediction of treatment responses and customization of pancreatic cancer treatment. Utilizing their predictive score, the model precisely forecasted the therapeutic outcome in patients new to treatment, achieving an impressive accuracy rate of 91.1% (Beutel et al., 2021).

One study suggested a mechanized, rapid-capacity, microflfluidic system for 3D organoid cultivation and examination, designed to offer both combinatorial and dynamic medicinal therapies to numerous cultures, facilitating instantaneous organoid analysis. The system was validated by individual, combination, and sequential drug screening for human-derived pancreatic tumor organoids. There were significant differences in the response to drug therapy in patient-derived organoids, and temporary modified drug therapy was found to be more effective than extracorporeal constant dose monotherapy or combination therapy (Schuster et al., 2020). The utility of microfluidic patient-derived organoids is proven by testing patient-derived organoids responses to several chemotherapies. Furthermore, the use of microfluidic organoid cultures serves to evaluate the efficacy of immunotherapy that combines NK cells with a new biological agent (Choi D. et al., 2023).

The US investigators have tested a combination of trametinib and hydroxychloroquine for organoids in patients with advanced pancreatic cancer. The results showed that this scheme had the highest inhibition rate of cancer cells in the patient’s tumor organoids among all the alternatives (Kinsey et al., 2019). The team executed an expandable prototype screening in pancreatic organoids affected by cystic fibrosis, employing various CFTR correctors and activators, and developed a gene therapy method based on mRNA in these organoids (Hohwieler et al., 2017). The research discovered that metformin reduced the expression of genes linked to cancer stemness in 3D co-cultures of PDAC organoids and PSC. The study suggests that metformin could provide a prospective method as a potent treatment for PDAC (Hahn et al., 2023). The single-organoid analysis successfully identified both resistant and invasive PDAC organoid clones, tailored to specific patient, therapy, medication, concentration, and time-specific levels (Le Compte et al., 2023). The utilization of patient-derived organoids (PDOs) for the characterization of therapeutic sensitivity and resistance represents a promising approach in precision medicine. PDOs are cultured in an extracellular matrix derived from basement membrane extract (BME). A study assessed the impact of different sources and batches of BME on proliferation, drug response to chemotherapy and targeted therapy, as well as gene expression in both mouse and human PDAC. The origin of BME does not alter the dose-response curve or drug test results of PDOs, further validating their reliability in characterizing drug sensitivity and resistance within the context of precision medicine (Lumibao et al., 2023).

With in-depth research, the swift advancement in organ-on-a-chip technology renders it an effective instrument for drug testing (Mo et al., 2020). The majority of co-culture methodologies for examining T2DM treatment encompass duct- and pancreatic islets-on-a-chip, as well as liver- and pancreatic islets-on-a-chip (Bauer et al., 2017; Shik Mun et al., 2019; Tao et al., 2022). One research created a three-dimensional chip for fat and pancreatic islets, designed for evaluating drugs in systemic metabolic disorders like T2DM (Xiu et al., 2019). Other study created a combined culture of small intestine and pancreatic islet on a chip, serving as an effective instrument for assessing glucose-induced fluctuations in endocrine hormones and for evaluating GLP-1 analogs and natural insulin in treating diabetes mellitus during antidiabetic drug screening (Nguyen et al., 2017).

### Application of pancreatic organoids in regenerative medicine of diabetes and other diseases

The creation of organoid cultures *in vitro* has garnered significant focus as a framework for researching regenerative medicine. Diabetes mellitus and PDAC stand as two significant destructive elements impacting the pancreas. T1DM, an autoimmune disorder, is marked by the depletion of pancreatic β-cells. Transplanting the pancreas or pancreatic islets represents the radical treatment. Generating β-like cells from stem cells presents a significant potential strategy in regenerative medicine. For individuals with insulin-dependent diabetes, substituting β-cells through pancreas or islet transplantation remains the sole prolonged treatment alternative. Glycemia can be restored when pancreatic organoids implanted in different preclinical diabetic models. Organoid transplantation and nanotechnology can avoid the immune attack by the host (Navarro-Tableros et al., 2020). In 2000, it was documented that the initial cells secreting insulin, originating from genetically altered ESCs, normalized blood glucose levels in mice with diabetes (Soria et al., 2000). In the following years, ESCs and iPSC’s therapeutic capabilities has been extensively tested and created of highly effective multi-stage differentiation methods (Dayem et al., 2019). In 2014, Rezania et. al described a protocol to differentiate human pluripotent stem cells into β cells *in vitro*, with β cells transplanted into mice reversing diabetes by pancreatic progenitors (Pagliuca et al., 2014; Rezania et al., 2014). Another study confirmed that islet organoid transplantation can improve diabetes reversal (Fonseca et al., 2023). Organoids that secrete insulin and are pre-vascularized, consisting of rat islet cells, hAECs, and human umbilical vein endothelial cells, demonstrate superior *in vitro* performance relative to native islets. The transplantation process for pre-vascularized islet organoids hastens the revascularization of the graft and improve blood glucose levels in immunodeficient diabetic mice (Wassmer et al., 2021). Hypoxia significantly contributes to the loss of islets in the initial phase following transplantation. Another study also demonstrated that hAEC can shield transplanted islets, protect from hypoxic damage, and improve β-cell implantation and islet revascularization, thus improving the ability of islets to reverse hyperglycemia (Lebreton et al., 2020). A magnetic sorting mechanism for the purification of microencapsulation in islets was found. Following the subcutaneous introduction of purified microencapsulated pancreatic islets into diabetic rats, their blood glucose levels reached a stable state for almost 17 weeks (Espona-Noguera et al., 2019).

**FIGURE 2:**
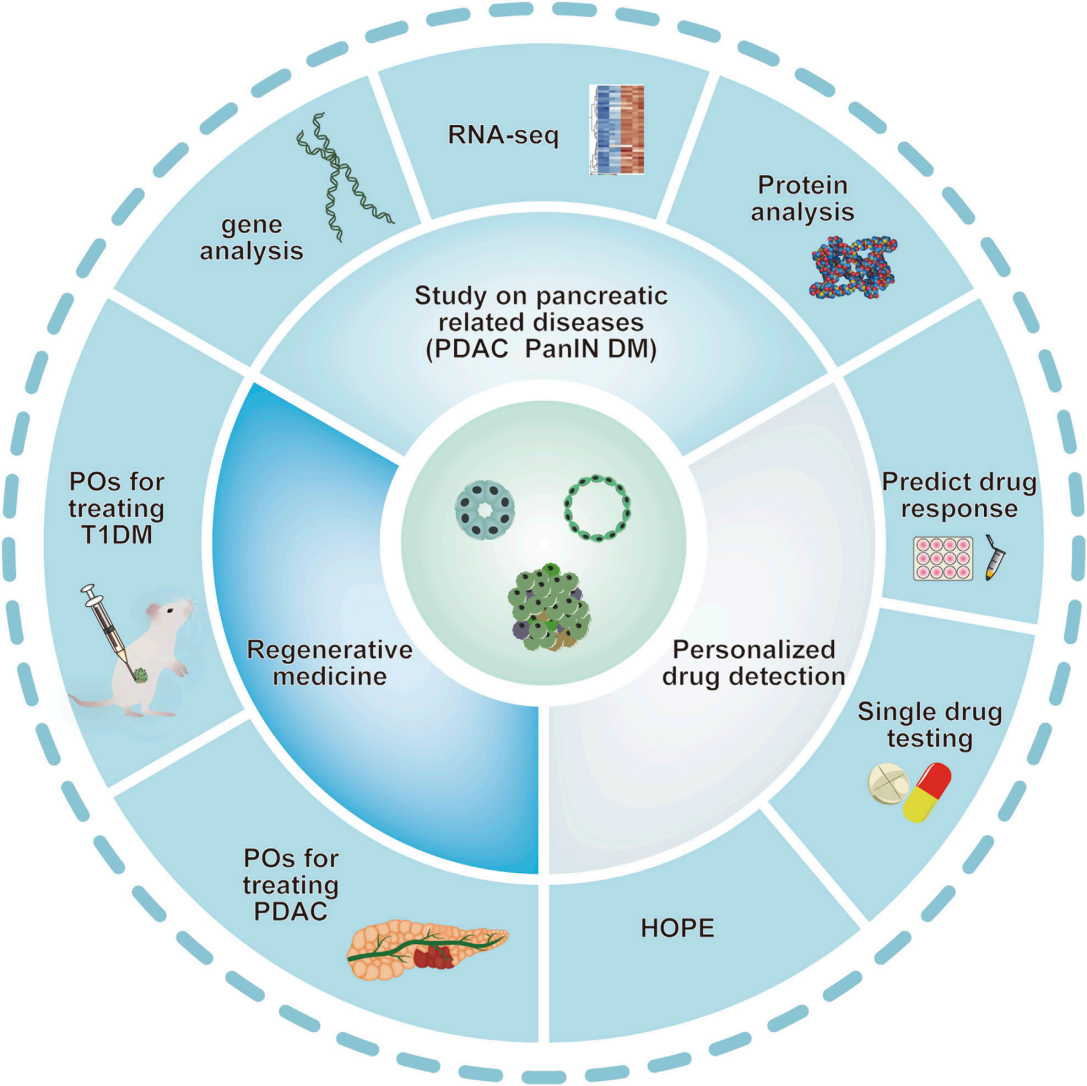
Various applications of pancreatic organoids. Schematic depiction of various applications of pancreatic organoids based on the studies mentioned above. Pancreatic organoids, including pancreatic exocrine and endocrine gland are mainly produced by hPSC and clinical primary ASCs. Organoid technology provides ideal models mimicking human pancreatic diseases. Pancreatic organoids have also provided a potential tool for personalized drug screening and application of in regenerative medicine. ASCs: adult stem cells; DM: diabetes mellitus; PanIN: pancreatic intraepithelial neoplasia; PDAC: pancreatic ductal adenocarcinoma; POs: pancreas organoids.

*In vitro*, human pancreas organoids have the ability to grow for several months while preserving their ductal structure, expression of biomarkers, and chromosomal wholeness. Upon being implanted into the pancreas of immunodeficient mice, human pancreas organoid xenografts endure for an extended period *in vivo* and exhibit no indications of developing tumors (Georgakopoulos et al., 2020). After transplanting human pluripotent stem cell-derived acinar/ductal organoids into immunodeficient mice, these organoids formed normal pancreatic duct and acinar tissue, similar to the pancreas of human fetus, without signs of tumor formation or transformation. The organoids exhibit the ultrastructural, widespread gene expression, and functional characteristics of the human pancreas in the dish (Hohwieler et al., 2017). (see Figure 2)

## Perspective and conclusion

The technology of pancreatic organoids has made significant progress in recent years. The construction of humanized pancreatic organoids has greatly enriched the donor sources, and promoted the in-depth research on pancreatic development and the identification and management of pancreatic disorders of cancer and non-cancer. However, there are still significant challenges in transitioning pancreatic organoid technology to clinical practice. How can pancreatic organoids cultured *in vitro* maximize the reproduction of the biological function of the pancreas? How to construct a complete pancreatic organoid with multiple cell types of endocrine and exocrine gland? And how to simplify the current complex culture system of pancreatic organoid to improve efficiency? Addressing these issues will undoubtedly promote the transformation of pancreatic organoids technology in precision medicine and regenerative medicine. So far, there is lack of a pancreatic organoid model containing both exocrine and endocrine glands, which hinders the physiological and pathophysiological studies of exocrine and endocrine glands of pancreas at the same time. Recently, the concept of assembloids (assembled organoids), also refer to the confused organoids, has been proposed (Pasca et al., 2022). Assembloids can be generated from organoids derived from different individuals, to create inter-individual assembloids, or different species, to create inter-species assembloids. Similarly, it is worth exploring whether it is possible to obtain the complete pancreatic organoid containing both exocrine and endocrine glands by the combination of exocrine organoid and endocrine organoid in the future study. In addition, the development of organoid on-chip also provides possibility for the co-culture of exocrine and endocrine organoid of pancreas together (Jalili-Firoozinezhad et al., 2021). Of course, microfluidic chips have brought new ideas for constructing composite organoid systems, and the application of this technology in pancreatic multi-lineage organoid systems is worth looking forward to. From a developmental perspective, organoid technology is still in its early stages, with the primary focus remaining on enhancing its biomimicry.

## References

[B1] Balboa D. Barsby T. Lithovius V. Saarimäki-Vire J. Omar-Hmeadi M. Dyachok O. (2022). Functional, metabolic and transcriptional maturation of human pancreatic islets derived from stem cells. Nat. Biotechnol. 40, 1042–1055. 10.1038/s41587-022-01219-z 35241836 PMC9287162

[B2] Bauer S. Wennberg Huldt C. Kanebratt K. P. Durieux I. Gunne D. Andersson S. (2017). Functional coupling of human pancreatic islets and liver spheroids on-a-chip: towards a novel human *ex vivo* type 2 diabetes model. Sci. Rep. 7, 14620. 10.1038/s41598-017-14815-w 29097671 PMC5668271

[B3] Beutel A. K. Schütte L. Scheible J. Roger E. Müller M. Perkhofer L. (2021). A prospective feasibility trial to challenge patient-derived pancreatic cancer organoids in predicting treatment response. Cancers (Basel) 13, 2539. 10.3390/cancers13112539 34064221 PMC8196829

[B4] Bhalerao N. Chakraborty A. Marciel M. P. Hwang J. Britain C. M. Silva A. D. (2023). ST6GAL1 sialyltransferase promotes acinar to ductal metaplasia and pancreatic cancer progression. JCI Insight 8, e161563. 10.1172/jci.insight.161563 37643018 PMC10619436

[B5] Bi H. Ye K. Jin S. (2020). Proteomic analysis of decellularized pancreatic matrix identifies collagen V as a critical regulator for islet organogenesis from human pluripotent stem cells. Biomaterials 233, 119673. 10.1016/j.biomaterials.2019.119673 31866049

[B6] Breunig M. Merkle J. Wagner M. Melzer M. K. Barth T. F. E. Engleitner T. (2021). Modeling plasticity and dysplasia of pancreatic ductal organoids derived from human pluripotent stem cells. Cell Stem Cell 28, 1105–1124.e19. 10.1016/j.stem.2021.03.005 33915078 PMC8461636

[B7] Bruin J. E. Erener S. Vela J. Hu X. Johnson J. D. Kurata H. T. (2014). Characterization of polyhormonal insulin-producing cells derived *in vitro* from human embryonic stem cells. Stem Cell Res. 12, 194–208. 10.1016/j.scr.2013.10.003 24257076

[B8] Bruin J. E. Rezania A. Xu J. Narayan K. Fox J. K. O’neil J. J. (2013). Maturation and function of human embryonic stem cell-derived pancreatic progenitors in macroencapsulation devices following transplant into mice. Diabetologia 56, 1987–1998. 10.1007/s00125-013-2955-4 23771205

[B9] Casamitjana J. Espinet E. Rovira M. (2022). Pancreatic organoids for regenerative medicine and cancer research. Front. Cell Dev. Biol. 10, 886153. 10.3389/fcell.2022.886153 35592251 PMC9110799

[B10] Chen K. Ma Y. Zhong X. Lan J. Long D. Tian X. (2023). Single-cell transcriptome profiling of primary tumors and paired organoids of pancreatobiliary cancer. Cancer Lett. 216586, 216586. 10.1016/j.canlet.2023.216586 38081505

[B11] Choi D. Gonzalez-Suarez A. M. Dumbrava M. G. Medlyn M. De Hoyos-Vega J. M. Cichocki F. (2023a). Microfluidic organoid cultures derived from pancreatic cancer biopsies for personalized testing of chemotherapy and immunotherapy. Adv. Sci. (Weinh) 11, e2303088. 10.1002/advs.202303088 38018486 PMC10837378

[B12] Choi J. Shin E. Lee J. Devarasou S. Kim D. Shin J. H. (2023b). Light-stimulated insulin secretion from pancreatic islet-like organoids derived from human pluripotent stem cells. Mol. Ther. 31, 1480–1495. 10.1016/j.ymthe.2023.03.013 36932674 PMC10188912

[B13] Clevers H. (2016). Modeling development and disease with organoids. Cell 165, 1586–1597. 10.1016/j.cell.2016.05.082 27315476

[B14] Criscimanna A. Speicher J. A. Houshmand G. Shiota C. Prasadan K. Ji B. (2011). Duct cells contribute to regeneration of endocrine and acinar cells following pancreatic damage in adult mice. Gastroenterology 141, e1451–e1462. 10.1053/j.gastro.2011.07.003 PMC4326039 21763240

[B15] Dayem A. A. Lee S. B. Kim K. Lim K. M. Jeon T. I. Cho S. G. (2019). Recent advances in organoid culture for insulin production and diabetes therapy: methods and challenges. BMB Rep. 52, 295–303. 10.5483/bmbrep.2019.52.5.089 30940326 PMC6549913

[B16] Demyan L. Habowski A. N. Plenker D. King D. A. Standring O. J. Tsang C. (2022). Pancreatic cancer patient-derived organoids can predict response to neoadjuvant chemotherapy. Ann. Surg. 276, 450–462. 10.1097/sla.0000000000005558 35972511 PMC10202108

[B17] Dilmen E. Orhon I. Jansen J. Hoenderop J. G. J. (2023). Advancements in kidney organoids and tubuloids to study (dys)function. Trends Cell Biol. 34, 299–311. 10.1016/j.tcb.2023.09.005 37865608

[B18] Du W. Wang J. Kuo T. Wang L. Mckimpson W. M. Son J. (2022). Pharmacological conversion of gut epithelial cells into insulin-producing cells lowers glycemia in diabetic animals. J. Clin. Invest. 132, e162720. 10.1172/jci162720 36282594 PMC9754100

[B19] Espona-Noguera A. Etxebarria-Elezgarai J. Saenz Del Burgo L. Cañibano-Hernández A. Gurruchaga H. Blanco F. J. (2019). Type 1 Diabetes Mellitus reversal via implantation of magnetically purified microencapsulated pseudoislets. Int. J. Pharm. 560, 65–77. 10.1016/j.ijpharm.2019.01.058 30742984

[B20] Fonseca L. M. Lebreton F. Wassmer C. H. Berishvili E. (2023). Generation of insulin-producing multicellular organoids. Methods Mol. Biol. 2592, 37–60. 10.1007/978-1-0716-2807-2_3 36507984

[B21] Fraunhoffer N. A. Abuelafia A. M. Bigonnet M. Gayet O. Roques J. Telle E. (2020). Evidencing a pancreatic ductal adenocarcinoma subpopulation sensitive to the proteasome inhibitor carfilzomib. Clin. Cancer Res. 26, 5506–5519. 10.1158/1078-0432.Ccr-20-1232 32669378

[B22] Fujii M. Matano M. Toshimitsu K. Takano A. Mikami Y. Nishikori S. (2018). Human intestinal organoids maintain self-renewal capacity and cellular diversity in niche-inspired culture condition. Cell Stem Cell 23, 787–793. 10.1016/j.stem.2018.11.016 30526881

[B23] Furuyama K. Kawaguchi Y. Akiyama H. Horiguchi M. Kodama S. Kuhara T. (2011). Continuous cell supply from a Sox9-expressing progenitor zone in adult liver, exocrine pancreas and intestine. Nat. Genet. 43, 34–41. 10.1038/ng.722 21113154

[B24] Gandhi S. De La Fuente J. Murad M. H. Majumder S. (2022). Chronic pancreatitis is a risk factor for pancreatic cancer, and incidence increases with duration of disease: a systematic review and meta-analysis. Clin. Transl. Gastroenterol. 13e00463, e00463. 10.14309/ctg.0000000000000463 PMC8963838 35142721

[B25] Georgakopoulos N. Prior N. Angres B. Mastrogiovanni G. Cagan A. Harrison D. (2020). Long-term expansion, genomic stability and *in vivo* safety of adult human pancreas organoids. BMC Dev. Biol. 20, 4. 10.1186/s12861-020-0209-5 32098630 PMC7043048

[B26] Gooch A. M. Chowdhury S. S. Zhang P. M. Hu Z. M. Westenfelder C. (2023). Significant expansion of the donor pool achieved by utilizing islets of variable quality in the production of allogeneic "Neo-Islets", 3-D organoids of Mesenchymal Stromal and islet cells, a novel immune-isolating biotherapy for Type I Diabetes. PLoS One 18, e0290460. 10.1371/journal.pone.0290460 37616230 PMC10449143

[B27] Hahn S. Oh B. J. Kim H. Han I. W. Shin S. H. Kim G. (2023). Anti-cancer effects of metformin in a 3D co-culture model of pancreatic ductal adenocarcinoma. Am. J. Cancer Res. 13, 1806–1825. 37293149 PMC10244103

[B28] Heaton E. S. Hu M. Liu T. Hui H. Tan Y. Ye K. (2023). Extracellular matrix-derived peptide stimulates the generation of endocrine progenitors and islet organoids from iPSCs. J. Tissue Eng. 14, 20417314231185858. 10.1177/20417314231185858 37435573 PMC10331343

[B29] Heiser P. W. Lau J. Taketo M. M. Herrera P. L. Hebrok M. (2006). Stabilization of beta-catenin impacts pancreas growth. Development 133, 2023–2032. 10.1242/dev.02366 16611688

[B30] Henning R. J. (2018). Type-2 diabetes mellitus and cardiovascular disease. Future Cardiol. 14, 491–509. 10.2217/fca-2018-0045 30409037

[B31] Hohwieler M. Illing A. Hermann P. C. Mayer T. Stockmann M. Perkhofer L. (2017). Human pluripotent stem cell-derived acinar/ductal organoids generate human pancreas upon orthotopic transplantation and allow disease modelling. Gut 66, 473–486. 10.1136/gutjnl-2016-312423 27633923 PMC5534761

[B32] Hossan M. S. Lin E. S. Riedl E. Stram A. Mehlhaff E. Koeppel L. (2023). Spatial alignment of organoids tracking subclonal chemotherapy resistance in pancreatic and ampullary cancer. Bioeng. (Basel) 10, 91. 10.3390/bioengineering10010091 PMC9854538 36671664

[B33] Hrvatin S. O’donnell C. W. Deng F. Millman J. R. Pagliuca F. W. Diiorio P. (2014). Differentiated human stem cells resemble fetal, not adult, β cells. Proc. Natl. Acad. Sci. U. S. A. 111, 3038–3043. 10.1073/pnas.1400709111 24516164 PMC3939927

[B34] Huang L. Desai R. Conrad D. N. Leite N. C. Akshinthala D. Lim C. M. (2021). Commitment and oncogene-induced plasticity of human stem cell-derived pancreatic acinar and ductal organoids. Cell Stem Cell 28, 1090–1104.e6. 10.1016/j.stem.2021.03.022 33915081 PMC8202734

[B35] Huang L. Holtzinger A. Jagan I. Begora M. Lohse I. Ngai N. (2015). Ductal pancreatic cancer modeling and drug screening using human pluripotent stem cell- and patient-derived tumor organoids. Nat. Med. 21, 1364–1371. 10.1038/nm.3973 26501191 PMC4753163

[B36] Huang X. Gu W. Zhang J. Lan Y. Colarusso J. L. Li S. (2023). Stomach-derived human insulin-secreting organoids restore glucose homeostasis. Nat. Cell Biol. 25, 778–786. 10.1038/s41556-023-01130-y 37106062 PMC10859913

[B37] Huch M. Bonfanti P. Boj S. F. Sato T. Loomans C. J. Van De Wetering M. (2013). Unlimited *in vitro* expansion of adult bi-potent pancreas progenitors through the Lgr5/R-spondin axis. Embo J. 32, 2708–2721. 10.1038/emboj.2013.204 24045232 PMC3801438

[B38] Ilegems E. Bryzgalova G. Correia J. Yesildag B. Berra E. Ruas J. L. (2022). HIF-1α inhibitor PX-478 preserves pancreatic β cell function in diabetes. Sci. Transl. Med. 14, eaba9112. 10.1126/scitranslmed.aba9112 35353540

[B39] Jalili-Firoozinezhad S. Miranda C. C. Cabral J. M. S. (2021). Modeling the human body on microfluidic chips. Trends Biotechnol. 39, 838–852. 10.1016/j.tibtech.2021.01.004 33581889

[B40] Kan C. Liu N. Zhang K. Wu D. Liang Y. Cai W. (2023). Global, regional, and national burden of pancreatic cancer, 1990-2019: results from the global burden of disease study 2019. Ann. Glob. Health 89, 33. 10.5334/aogh.4019 37252335 PMC10215993

[B41] Kao D. I. Lacko L. A. Ding B. S. Huang C. Phung K. Gu G. (2015). Endothelial cells control pancreatic cell fate at defined stages through EGFL7 signaling. Stem Cell Rep. 4, 181–189. 10.1016/j.stemcr.2014.12.008 PMC4325230 25601205

[B42] Kinsey C. G. Camolotto S. A. Boespflug A. M. Guillen K. P. Foth M. Truong A. (2019). Publisher Correction: protective autophagy elicited by RAF→MEK→ERK inhibition suggests a treatment strategy for RAS-driven cancers. Nat. Med. 25, 861. 10.1038/s41591-019-0433-3 30918364

[B43] Kopp J. L. Dubois C. L. Schaffer A. E. Hao E. Shih H. P. Seymour P. A. (2011). Sox9+ ductal cells are multipotent progenitors throughout development but do not produce new endocrine cells in the normal or injured adult pancreas. Development 138, 653–665. 10.1242/dev.056499 21266405 PMC3026412

[B44] Kroon E. Martinson L. A. Kadoya K. Bang A. G. Kelly O. G. Eliazer S. (2008). Pancreatic endoderm derived from human embryonic stem cells generates glucose-responsive insulin-secreting cells *in vivo* . Nat. Biotechnol. 26, 443–452. 10.1038/nbt1393 18288110

[B45] Lancaster M. A. Knoblich J. A. (2014). Organogenesis in a dish: modeling development and disease using organoid technologies. Science 345, 1247125. 10.1126/science.1247125 25035496

[B46] Lebreton F. Bellofatto K. Wassmer C. H. Perez L. Lavallard V. Parnaud G. (2020). Shielding islets with human amniotic epithelial cells enhances islet engraftment and revascularization in a murine diabetes model. Am. J. Transpl. 20, 1551–1561. 10.1111/ajt.15812 32031745

[B47] Lebreton F. Lavallard V. Bellofatto K. Bonnet R. Wassmer C. H. Perez L. (2019). Insulin-producing organoids engineered from islet and amniotic epithelial cells to treat diabetes. Nat. Commun. 10, 4491. 10.1038/s41467-019-12472-3 31582751 PMC6776618

[B48] Le Compte M. De La Hoz E. C. Peeters S. Fortes F. R. Hermans C. Domen A. (2023). Single-organoid analysis reveals clinically relevant treatment-resistant and invasive subclones in pancreatic cancer. NPJ Precis. Oncol. 7, 128. 10.1038/s41698-023-00480-y 38066116 PMC10709344

[B49] Lee M. G. Ohana E. Park H. W. Yang D. Muallem S. (2012). Molecular mechanism of pancreatic and salivary gland fluid and HCO3 secretion. Physiol. Rev. 92, 39–74. 10.1152/physrev.00011.2011 22298651 PMC3667394

[B50] Lin J. C. Liu T. P. Yang P. M. (2020). CDKN2A-Inactivated pancreatic ductal adenocarcinoma exhibits therapeutic sensitivity to paclitaxel: a bioinformatics study. J. Clin. Med. 9, 4019. 10.3390/jcm9124019 33322698 PMC7763913

[B51] Loomans C. J. M. Williams Giuliani N. Balak J. Ringnalda F. Van Gurp L. Huch M. (2018). Expansion of adult human pancreatic tissue yields organoids harboring progenitor cells with endocrine differentiation potential. Stem Cell Rep. 10, 712–724. 10.1016/j.stemcr.2018.02.005 PMC5918840 29539434

[B52] Lumibao J. C. Okhovat S. R. Peck K. L. Lin X. Lande K. Yomtoubian S. (2023). The effect of extracellular matrix on the precision medicine utility of pancreatic cancer patient-derived organoids. JCI Insight 9, e172419. 10.1172/jci.insight.172419 PMC10906458 38051586

[B53] Lyu X. Rowley M. J. Kulik M. J. Dalton S. Corces V. G. (2023). Regulation of CTCF loop formation during pancreatic cell differentiation. Nat. Commun. 14, 6314. 10.1038/s41467-023-41964-6 37813869 PMC10562423

[B54] Mamidi A. Prawiro C. Seymour P. A. De Lichtenberg K. H. Jackson A. Serup P. (2018). Mechanosignalling via integrins directs fate decisions of pancreatic progenitors. Nature 564, 114–118. 10.1038/s41586-018-0762-2 30487608

[B55] Mansour A. A. Gonçalves J. T. Bloyd C. W. Li H. Fernandes S. Quang D. (2018). An *in vivo* model of functional and vascularized human brain organoids. Nat. Biotechnol. 36, 432–441. 10.1038/nbt.4127 29658944 PMC6331203

[B56] Marsee A. Roos F. J. M. Verstegen M. M. A. Gehart H. De Koning E. Lemaigre F. (2021). Building consensus on definition and nomenclature of hepatic, pancreatic, and biliary organoids. Cell Stem Cell 28, 816–832. 10.1016/j.stem.2021.04.005 33961769 PMC11699540

[B57] Matsuura T. Maru Y. Izumiya M. Hoshi D. Kato S. Ochiai M. (2020). Organoid-based *ex vivo* reconstitution of Kras-driven pancreatic ductal carcinogenesis. Carcinogenesis 41, 490–501. 10.1093/carcin/bgz122 31233118

[B58] Maxwell K. G. Augsornworawat P. Velazco-Cruz L. Kim M. H. Asada R. Hogrebe N. J. (2020). Gene-edited human stem cell-derived β cells from a patient with monogenic diabetes reverse preexisting diabetes in mice. Sci. Transl. Med. 12, eaax9106. 10.1126/scitranslmed.aax9106 32321868 PMC7233417

[B59] Merz S. Breunig M. Melzer M. K. Heller S. Wiedenmann S. Seufferlein T. (2023). Single-cell profiling of GP2-enriched pancreatic progenitors to simultaneously create acinar, ductal, and endocrine organoids. Theranostics 13, 1949–1973. 10.7150/thno.78323 37064874 PMC10091881

[B60] Mo S. J. Lee J. H. Kye H. G. Lee J. M. Kim E. J. Geum D. (2020). A microfluidic gradient device for drug screening with human iPSC-derived motoneurons. Analyst 145, 3081–3089. 10.1039/c9an02384d 32150196

[B61] Murtaugh L. C. Law A. C. Dor Y. Melton D. A. (2005). Beta-catenin is essential for pancreatic acinar but not islet development. Development 132, 4663–4674. 10.1242/dev.02063 16192304

[B62] Navarro-Tableros V. Gomez Y. Brizzi M. F. Camussi G. (2020). Generation of human stem cell-derived pancreatic organoids (POs) for regenerative medicine. Adv. Exp. Med. Biol. 1212, 179–220. 10.1007/5584_2019_340 31025308

[B63] Nguyen D. T. Van Noort D. Jeong I. K. Park S. (2017). Endocrine system on chip for a diabetes treatment model. Biofabrication 9, 015021. 10.1088/1758-5090/aa5cc9 28222044

[B64] Pagliuca F. W. Millman J. R. Gürtler M. Segel M. Van Dervort A. Ryu J. H. (2014). Generation of functional human pancreatic β cells *in vitro* . Cell 159, 428–439. 10.1016/j.cell.2014.09.040 25303535 PMC4617632

[B65] Pan F. C. Bankaitis E. D. Boyer D. Xu X. Van De Casteele M. Magnuson M. A. (2013). Spatiotemporal patterns of multipotentiality in Ptf1a-expressing cells during pancreas organogenesis and injury-induced facultative restoration. Development 140, 751–764. 10.1242/dev.090159 23325761 PMC3557774

[B66] Pasca S. P. Arlotta P. Bateup H. S. Camp J. G. Cappello S. Gage F. H. (2022). A nomenclature consensus for nervous system organoids and assembloids. Nature 609, 907–910. 10.1038/s41586-022-05219-6 36171373 PMC10571504

[B67] Pasca Di Magliano M. Biankin A. V. Heiser P. W. Cano D. A. Gutierrez P. J. Deramaudt T. (2007). Common activation of canonical Wnt signaling in pancreatic adenocarcinoma. PLoS One 2, e1155. 10.1371/journal.pone.0001155 17982507 PMC2048934

[B68] Peery A. F. Crockett S. D. Murphy C. C. Lund J. L. Dellon E. S. Williams J. L. (2019). Burden and cost of gastrointestinal, liver, and pancreatic diseases in the United States: update 2018. Gastroenterology 156, 254–272. 10.1053/j.gastro.2018.08.063 30315778 PMC6689327

[B69] Picozzi V. J. (2024). Pancreatic cancer: new approaches to drug therapy. Int. J. Surg. 2024. 10.1097/JS9.0000000000000877 PMC11486970 38573111

[B70] Ramzy A. Thompson D. M. Ward-Hartstonge K. A. Ivison S. Cook L. Garcia R. V. (2021). Implanted pluripotent stem-cell-derived pancreatic endoderm cells secrete glucose-responsive C-peptide in patients with type 1 diabetes. Cell Stem Cell 28, 2047–2061.e5. 10.1016/j.stem.2021.10.003 34861146

[B71] Rezania A. Bruin J. E. Arora P. Rubin A. Batushansky I. Asadi A. (2014). Reversal of diabetes with insulin-producing cells derived *in vitro* from human pluripotent stem cells. Nat. Biotechnol. 32, 1121–1133. 10.1038/nbt.3033 25211370

[B72] Rohayem J. Ehlers C. Wiedemann B. Holl R. Oexle K. Kordonouri O. (2011). Diabetes and neurodegeneration in Wolfram syndrome: a multicenter study of phenotype and genotype. Diabetes Care 34, 1503–1510. 10.2337/dc10-1937 21602428 PMC3120194

[B73] Schuster B. Junkin M. Kashaf S. S. Romero-Calvo I. Kirby K. Matthews J. (2020). Automated microfluidic platform for dynamic and combinatorial drug screening of tumor organoids. Nat. Commun. 11, 5271. 10.1038/s41467-020-19058-4 33077832 PMC7573629

[B74] Seino T. Kawasaki S. Shimokawa M. Tamagawa H. Toshimitsu K. Fujii M. (2018). Human pancreatic tumor organoids reveal loss of stem cell niche factor dependence during disease progression. Cell Stem Cell 22, 454–467. 10.1016/j.stem.2017.12.009 29337182

[B75] Shang L. Hua H. Foo K. Martinez H. Watanabe K. Zimmer M. (2014). β-cell dysfunction due to increased ER stress in a stem cell model of Wolfram syndrome. Diabetes 63, 923–933. 10.2337/db13-0717 24227685 PMC3931392

[B76] Shapiro A. M. J. Thompson D. Donner T. W. Bellin M. D. Hsueh W. Pettus J. (2021). Insulin expression and C-peptide in type 1 diabetes subjects implanted with stem cell-derived pancreatic endoderm cells in an encapsulation device. Cell Rep. Med. 2, 100466. 10.1016/j.xcrm.2021.100466 35028608 PMC8714853

[B77] Sharma A. Sances S. Workman M. J. Svendsen C. N. (2020). Multi-lineage human iPSC-derived platforms for disease modeling and drug discovery. Cell Stem Cell 26, 309–329. 10.1016/j.stem.2020.02.011 32142662 PMC7159985

[B78] Shiihara M. Ishikawa T. Saiki Y. Omori Y. Hirose K. Fukushige S. (2021). Development of a system combining comprehensive genotyping and organoid cultures for identifying and testing genotype-oriented personalised medicine for pancreatobiliary cancers. Eur. J. Cancer 148, 239–250. 10.1016/j.ejca.2021.01.047 33752134

[B79] Shik Mun K. Arora K. Huang Y. Yang F. Yarlagadda S. Ramananda Y. (2019). Patient-derived pancreas-on-a-chip to model cystic fibrosis-related disorders. Nat. Commun. 10, 3124. 10.1038/s41467-019-11178-w 31311920 PMC6635497

[B80] Siegel R. Miller K. Fuchs H. Jemal A. (2022). Cancer statistics, 2022. CA a cancer J. Clin. 72, 7–33. 10.3322/caac.21708 35020204

[B81] Simsek S. Zhou T. Robinson C. L. Tsai S. Y. Crespo M. Amin S. (2016). Modeling cystic fibrosis using pluripotent stem cell-derived human pancreatic ductal epithelial cells. Stem Cells Transl. Med. 5, 572–579. 10.5966/sctm.2015-0276 27034411 PMC4835252

[B82] Sinha S. Fu Y. Y. Grimont A. Ketcham M. Lafaro K. Saglimbeni J. A. (2017). PanIN neuroendocrine cells promote tumorigenesis via neuronal cross-talk. Cancer Res. 77, 1868–1879. 10.1158/0008-5472.Can-16-0899-t 28386018 PMC5471615

[B83] Soria B. Roche E. Berná G. León-Quinto T. Reig J. A. Martín F. (2000). Insulin-secreting cells derived from embryonic stem cells normalize glycemia in streptozotocin-induced diabetic mice. Diabetes 49, 157–162. 10.2337/diabetes.49.2.157 10868930

[B84] Takebe T. Sekine K. Kimura M. Yoshizawa E. Ayano S. Koido M. (2017). Massive and reproducible production of liver buds entirely from human pluripotent stem cells. Cell Rep. 21, 2661–2670. 10.1016/j.celrep.2017.11.005 29212014

[B85] Takeuchi K. Tabe S. Takahashi K. Aoshima K. Matsuo M. Ueno Y. (2023). Incorporation of human iPSC-derived stromal cells creates a pancreatic cancer organoid with heterogeneous cancer-associated fibroblasts. Cell Rep. 42, 113420. 10.1016/j.celrep.2023.113420 37955987

[B86] Tao T. Deng P. Wang Y. Zhang X. Guo Y. Chen W. (2022). Microengineered multi-organoid system from hiPSCs to recapitulate human liver-islet Axis in normal and type 2 diabetes. Adv. Sci. (Weinh) 9, e2103495. 10.1002/advs.202103495 34951149 PMC8844474

[B87] Teo A. K. Tsuneyoshi N. Hoon S. Tan E. K. Stanton L. W. Wright C. V. (2015). PDX1 binds and represses hepatic genes to ensure robust pancreatic commitment in differentiating human embryonic stem cells. Stem Cell Rep. 4, 578–590. 10.1016/j.stemcr.2015.02.015 PMC4400640 25843046

[B88] Van De Casteele M. Leuckx G. Baeyens L. Cai Y. Yuchi Y. Coppens V. (2013). Neurogenin 3+ cells contribute to β-cell neogenesis and proliferation in injured adult mouse pancreas. Cell Death Dis. 4, e523. 10.1038/cddis.2013.52 23470530 PMC3613830

[B89] Wang D. Wang J. Bai L. Pan H. Feng H. Clevers H. (2020a). Long-term expansion of pancreatic islet organoids from resident Procr+ progenitors. Cell 180, 1198–1211. 10.1016/j.cell.2020.02.048 32200801

[B93] Wassmer C. H. Lebreton F. Bellofatto K. Perez L. Cottet-Dumoulin D. Andres A. (2021). Bio-engineering of pre-vascularized islet organoids for the treatment of type 1 diabetes. Transpl. Int. 35, 10214. 10.3389/ti.2021.10214 35185372 PMC8842259

[B94] Xie R. Everett L. Lim H. Patel N. Schug J. Kroon E. (2013). Dynamic chromatin remodeling mediated by polycomb proteins orchestrates pancreatic differentiation of human embryonic stem cells. Cell stem Cell 12, 224–237. 10.1016/j.stem.2012.11.023 23318056 PMC3619036

[B95] Xinaris C. Brizi V. Remuzzi G. (2015). Organoid models and applications in biomedical research. Nephron 130, 191–199. 10.1159/000433566 26112599

[B96] Xiu Y. Xiu-Li Z. Yong L. Li-Chao L. Wei-Jie Z. Bing-Cheng L. (2019). Establishment of 3D organ chip for multiplexed assessment of type 2 diabetes drugs. Prog. Biochem. Biophysics 46, 620–630. 10.16476/j.pibb.2018.0337

[B97] Xu X. D’hoker J. Stangé G. Bonné S. De Leu N. Xiao X. (2008). Beta cells can be generated from endogenous progenitors in injured adult mouse pancreas. Cell 132, 197–207. 10.1016/j.cell.2007.12.015 18243096

[B98] Xue L. Wang H. He Y. Sui M. Li H. Mei L. (2022). Incidence and risk factors of diabetes mellitus in the Chinese population: a dynamic cohort study. BMJ Open 12, e060730. 10.1136/bmjopen-2021-060730 PMC9680191 36410801

[B99] Yumi I. Scott A S. Jeffery S T. (2023). Editorial: study of pancreatic islets based on human models to understand pathogenesis of diabetes. Front. Endocrinol. (Lausanne) 13. 10.3389/fendo.2022.1128653 PMC9875287 36714557

